# Combined Expression of IFN-γ, IL-17, and IL-4 mRNA by Recall PBMCs Moderately Discriminates Active Tuberculosis from Latent *Mycobacterium tuberculosis* Infection in Patients with Miscellaneous Inflammatory Underlying Conditions

**DOI:** 10.3389/fimmu.2016.00239

**Published:** 2016-06-14

**Authors:** Laura E. Savolainen, Anu Kantele, Aija Knuuttila, Liana Pusa, Riitta Karttunen, Heikki Valleala, Tamara Tuuminen

**Affiliations:** ^1^Department of Bacteriology and Immunology, Haartman Institute, University of Helsinki, Helsinki, Finland; ^2^Department of Medicine, Division of Infectious Diseases, Helsinki University Central Hospital, Helsinki, Finland; ^3^Department of Medicine, Institute of Clinical Medicine, University of Helsinki, Helsinki, Finland; ^4^Heart and Lung Center, Helsinki University Central Hospital, Helsinki, Finland; ^5^Länsi-Uusimaa Hospital, Tammisaari, Finland; ^6^Laboratory Division (HUSLAB), Helsinki University Central Hospital, Helsinki, Finland; ^7^Department of Medicine, Division of Rheumatology, Helsinki University Central Hospital, Helsinki, Finland; ^8^Eastern Finland Laboratory Centre Joint Authority Enterprise, Mikkeli, Finland

**Keywords:** IFN-γ, IL-17, IL-4, mRNA, active TB, LTBI

## Abstract

New biomarkers are needed for discriminating active tuberculosis (TB) from latent TB infection (LTBI), especially in vulnerable groups representing the major diagnostic challenge. This pilot study was carried out to explore the diagnostic potential of selected genes, *IFN-*γ, *IL-17*, *IL-4*, and *FoxP3*, associated with TB immunity and immunopathology. IFN-γ, IL-17, IL-4, and FoxP3 mRNA expression levels were measured by quantitative reverse transcription PCR (RT-qPCR) from antigen-stimulated peripheral blood mononuclear cells of patients with active TB (*n* = 25); patients with miscellaneous inflammatory disorders and concomitant LTBI (*n* = 20), rheumatoid arthritis (RA) being the most predominant in the group (*n* = 11); and in healthy Bacillus Calmette–Guérin (BCG) vaccinees (*n* = 8). While the levels of FoxP3 mRNA did not differ between the tested groups, the cumulative expression levels of purified protein derivative-stimulated IFN-γ, IL-17, and IL-4 mRNAs were found to distinguish active TB from the whole group of LTBI with 48% sensitivity and 85% specificity. When restricting the LTBI group to RA cases only, the sensitivity was 56% and specificity 100%. When interpreting the result as positive in at least one of the mRNAs IFN-γ, IL-17, or IL-4, sensitivity of 64% and specificities of 75% (heterogeneous group of LTBI) or 100% (LTBI with RA) were achieved. Moderate discrimination of active TB from LTBI with miscellaneous inflammatory underlying conditions by using combined quantitative expression of IFN-γ, IL-17, and IL-4 mRNA seems not to be of high diagnostic potential.

## Introduction

T cell-mediated immunity through the Th1-arm of the immunologic system serves an important function in controlling tuberculosis (TB). Almost everyone infected with *Mycobacterium tuberculosis (Mtb)* produces IFN-γ and TNF-α, yet these cytokines cannot confer protection against clinical disease (1).

T cell-based IFN-γ release assays (IGRAs) developed a decade ago are the first diagnostic tests conceived after the century-old tuberculin skin test for identification of contact with *Mtb* (2). Although not capable of discriminating active TB from latent TB infection (LTBI), IGRAs are used in areas with low TB incidence as a supplemental method in subjects with symptoms suggestive of TB. A negative IGRA test in an immunocompetent patient indicates that active TB is improbable. (3) From the diagnostic point of view, patients with miscellaneous inflammatory musculoskeletal disorders responding poorly to conventional therapy present a great diagnostic challenge. In these patients, LTBI should be excluded before the commencement of the so-called biological therapy with, e.g., TNF-α blockers, due to the risk of TB reactivation (4).

The diagnostic potential of T cell subsets other than Th1 and its markers is not yet fully exploited. For example, the recently described Th17 subset (5) producing proinflammatory cytokine IL-17 promotes chemokine expression and the recruitment of neutrophils and stimulates T cells producing IFN-γ in the lungs. This cytokine is important in early granuloma formation, i.e., the protective mechanism to wall off an infection (6). However, when neutrophil recruitment gets too extensive and IL-17 is secreted in excess, the fine balance is shifted to “overwhelmed” immune response, resulting in tissue damage and necrosis inside the granuloma (7).

Regulatory T cells (Tregs) expressing the forkhead box P3 (FoxP3) serve to limit the effector response to the infectious agent, which may result in a failure of infection control. These cells have proved to minimize tissue damage and immunopathological effects caused by effector mechanisms. (8) For example, in a murine model of TB, Tregs delayed the recruitment of *Mtb*-specific effector T cells to the lungs, thus impairing immune protection (9). Indeed, higher levels of Tregs in peripheral blood and the lungs of patients with active TB compared to healthy controls were described (10, 11).

Th2 cells have a distinct role in the pathogenesis of TB. The excessive production of IL-4 may attenuate the Th1 response and lead to severe immunopathological consequences (12). Elevated levels of IL-4 mRNA have been detected from peripheral blood mononuclear cells (PBMCs) of patients with active TB (13) and those LTBI alike (14). On the other hand, IL-4δ2, which is an antagonist of IL-4 and a truncated variant of IL-4, has proved elevated in LTBI and in cured TB (13, 15, 16). The ratio of IL-4 to IL-4δ2 has been suggested to function as a reliable marker of treatment efficacy (13, 16).

This study was undertaken to explore the diagnostic value of *IFN-*γ, *IL-4*, *IL-17*, and *FoxP3* gene expression levels. We set out to investigate whether the quantitative expression of these biomarkers differs between groups with active TB and LTBI.

## Materials and Methods

### Ethical Statements

The study protocol was approved by the Southwest Finland district Ethical Committee (DroNo 47/180/2009) and by Helsinki and Uusimaa Hospital district (149/2010). All patients provided written informed consent.

### Study Subjects

Nine milliliters of whole heparinized blood was collected from 25 patients with active TB group, 20 with LTBI (LTBI group), and 8 healthy volunteers with a history of Bacillus Calmette–Guérin vaccination in their childhood (BCG group). Subjects with LTBI were further subdivided. The rheumatoid arthritis (RA) subgroup included the patients with RA (*n* = 11). The MISC subgroup (*n* = 9) includes the patients with miscellaneous inflammatory conditions (one of each: vasculitis, ankylosing spondylitis, myiasis, mycoplasma infection, streptococcal skin infection, unknown skin infection, tonsillitis, COPD, and weakness of skin sensation). IGRA tests were performed as a diagnostic work-up in LTBI group, and active TB was excluded in all. In the TB group, 22 patients had been diagnosed with pulmonary TB and 3 with extrapulmonary TB. Only one pulmonary TB patient was diagnosed on the basis of clinical presentation, radiological findings consistent with TB, and a good response to anti-TB treatment. All other TB patients were diagnosed by *Mtb* isolation. The blood samples of all TB patients were drawn not more than 2 weeks after initiation of anti-TB treatment (median: 7 days, range: 0–14 days). LTBI diagnosis was composed on the basis of a positive IGRA result and a history of TB exposure, with neither radiological nor clinical signs of active TB. In the BCG group, LTBI was excluded by negative IGRA result. The demographic and clinical characteristics of the study subjects are presented in Table 1. Previous treatment schemes for RA are shown in Table 2. All enrolled patients and healthy subjects were HIV negative.

**Table 1:** **Clinical and demographical data of enrolled subjects**.

Patient groups	*n*	Age range (mean)	Female (*n*) %	AFB pos. (*n*) %	Culture pos (*n*) %	IGRA pos (*n*) %
Active TB (TB)	25	24–87 (44)	10 (40)	19 (76)	24 (96)	n/d
Active TB, pulmonary (TB)	22	26–87 (45)	8 (36)	19 (86)	21 (95)	n/d
Active TB, extrapulmonary (TB)	3	24–61 (36)	2 (67)	0 (0)	3 (100)	n/d
Latently infected (LTBI)	20	34–77 (61)	10 (50)	n/d	n/d	20 (100)
Rheumatic diseases^a^	13	49–77 (65)	8 (62)	n/d	n/d	13 (100)
Miscellaneous conditions^b^	7	34–64 (56)	2 (29)	n/d	n/d	7 (100)
Healthy BCG vaccinees (BCG)	8	25–50 (34)	6 (75)	n/d	n/d	0 (0)

*n/d, not defined*.

*^a^RA, rheumatic arthritis (*n* = 10); JRA, juvenile rheumatic arthritis (*n* = 1), vasculitis (*n* = 1), SPA ankylosing spondylitis (*n* = 1)*.

*^b^Infection other than TB (*n* = 5), COPD (*n* = 1), weakness of skin sensation (*n* = 1)*.

**Table 2:** **Characteristics of LTBI patients with a rheumatic disease**.

Gender	Diagnosis	Disease duration (years)	Biological medicine (months)	Prednisone dose (mg/day)	DMARDs^a^	CRP^b^ mg/l	ESR^c^ mm/h
M	RA^d^ (M05.8)	26	Etanercept (32)	5	Mtx^e^, SASP^f^, and HCQ^g^	3	17
F	RA (M06.0)	28	Etanercept (59)	5	–	11	36
M	RA (M05.8)	16	–^k^	10	Mtx	87	39
F	RA (M06.0)	9	Etanercept (1)	–	–	10	8
F	RA (M05.8)	6	–	5	Mtx, leflunomide, and HCQ	<3	2
F	RA (M05.8)	20	Rituximab (12)	5	–	29	34
F	JRA^h^ (M08.0)	64	Rituximab (23)	7.5	–	18	13
F	RA (M05.8)	8	–	5	Leflunomide	71	41
F	RA (M05.8)	8	–	–	SASP	13	22
M	RA (M05.8)	7	Rituximab (24)	5	Mtx and HCQ	72	57
M	Vascultis (L95.8)	1	–	5	HCQ	7	5
M	RA (M05.8)	18	–	7.5	Mtx and CyA^i^	<3	26
F	SPA^j^ (M45)	4	Golimumab (1)	–	Mtx and SASP	4	12

*^a^Disease-modifying antirheumatic drug*.

*^b^C-reactive protein*.

*^c^Erythrosyte sedimentation rate*.

*^d^Rheumatoid arthritis*.

*^e^Methotrexate*.

*^f^Sulfasalazine*.

*^g^Hydroxychloroquine*.

*^h^JRA, juvenile rheumatoid arthritis*.

*^i^Cyclosporine*.

*^j^SPA ankylosing spondylitis*.

*^k^Etanercept stopped 9 months earlier (used for 21 months)*.

### Quantitative Reverse Transcription PCR

Peripheral blood mononuclear cells were isolated with Ficoll paque (Amersham Biosciences AB, Uppsala, Sweden) centrifugation and stored in liquid nitrogen until use or stimulated immediately. A total of the cells were incubated in RPMI-10% FCS (Sigma–Aldrich, Saint Louis, MO, USA) with purified protein derivative (PPD) [Statens serum institute (SSI)] (10 μg/ml), pool of culture filtrate protein 10 (CFP-10), and early secretory antigenic target (ESAT-6) peptides (Oxford Immunotec, Oxford, UK) and without antigen for 22 h (17, 18) at 37°C, 5%CO^2^ and lysed with TRI Reagent^®^ (Molecular research center, Inc., Cincinnati, OH, USA). mRNA was extracted with RNeasy mini kit (Qiagen, Dusseldorf, Germany) according to manufacturer’s instructions and transcribed using AMV reverse transcriptase (New England Biolabs, Ipswich, England), Oligo(dT)_23_ primers (Sigma–Aldrich), and dNTP Mix (Thermo Scientific, Waltham, MA, USA) in a final volume of 20 μl. RiboLock RNase Inhibitor was used (thermo scientific). Amplification was performed in a total volume of 20 μl using the Power SYBR^®^ Green PCR Master Mix (Life Technologies Ltd., Paisley, UK) as instructed. The validated (19) housekeeping gene, human ribosomal protein (*HuPO*), was used to normalize the mRNA values. The primers used included *HuPO* forward, 5′-GCAATGTTGCCAGTGTCTGT-3′, *HuPO* reverse, 5′-GCCTTGACCTTTTCAGCAAG-3′; *IFN-*γ forward, 5′-ATTCGGTAACTGACTTGAATGTCC-3′, *IFN-*γ reverse, 5′-CTCTTCGACCTCGAAACAGC-3′ (20); *IL-4* forward, 5′-CGA GTT GAC CGT AAC AGA CAT-3′, *IL-4* reverse, 5′-CGT CTT TAG CCT TTC CAA GAAG-3′; *IL4*δ*2* forward, 5′-CAGAGCAGAAGAACACAACTG, *IL4*δ*2* reverse, 5′-GTCTTTAGCCTTTCCAAGAAG-3′ (15); *IL-17* forward, 5′-GGA CTG TGA TGG TCA ACC TGA-3′, *IL-17* reverse, 5′-TCA TGT GGT AGT CCA CGT TCC-3′ (21); *FoxP3* forward, 5′-ACCTGGAAGAACGCCATC-3′, *FOXP3* reverse, 5′-TGTTCGTCCATCCTCCTTTC-3′ (22). qPCR analysis was performed with iCycleriQ™ Real-Time PCR detection system (Biorad, Hercules, CA, USA). Forty cycles were run with initial melting step of 5 min at 95°C followed by cycles of 45 s at 95°C, 45 s at 60°C, and 45 s at 72°C. Specificity of the primers was ensured once with gel electrophoresis and later with melting curve analysis. The results were analyzed with comparative *C*_t_ method 2^ΔΔCt^ (23).

### Statistical Analysis

The data were analyzed with GraphPad Prism version 6.0 (GraphPad Software, Inc., San Diego, CA, USA.). Mann–Whitney *U*-test was used for testing the differences between the groups; *p* < 0.05 was considered significant. Cutoff points with highest combined sensitivity and specificity were calculated with receiver operating-characteristic (ROC) curves and discriminative ability with area under the curves (AUCs).

## Results

### Selection of Genes for Further Evaluation

We first tested the ability of PPD to stimulate different genes and the discriminative power of selected genes by analyzing five samples from each study group. In these pilot experiments, mRNA expression levels of IFN-γ (*p* = 0.095), IL-17 (*p* = 0.1032), IL-4 (*p* = 0.087), and FoxP3 (*p* = 0.286) were higher in the TB than in the LTBI group (data not shown).

### CFP-10 and ESAT-6 Peptide Pools As Gene Expression Stimulators

**Figure 1:**
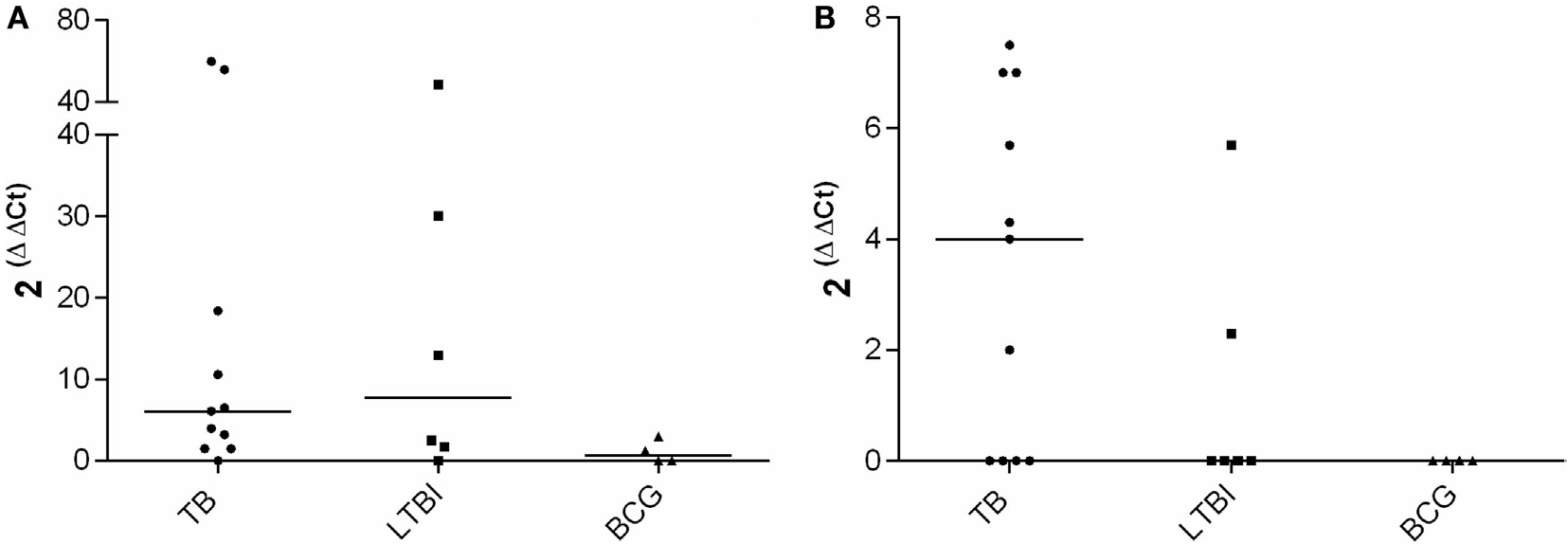
**CFP-10-ESAT-6 peptide pool stimulated mRNA expression**. Fold increase in IFN-γ **(A)** and IL-4 **(B)** expression in TB (*n* = 11), LTBI (*n* = 6) and BCG (*n* = 4) groups. Medians of the groups are shown with horizontal bars.

mRNA expression levels of IFN-γ, IL-17, IL-4, and FoxP3 were analyzed after stimulation with a pool of peptides derived from CFP-10 and ESAT-6. Peptides stimulated IFN-γ and IL-4 mRNA expression, but difference was not observed between the medians of the IFN-γ expression in the TB and the LTBI groups (Figure 1). In the TB group, the median of IL-4 mRNA expression appeared higher than that in the LTBI group, yet the difference did not prove statistically significant (Figure 1). The peptide antigen stimulation resulted in the expression of IL-4 mRNA in two LTBI patients also, and those were diagnosed with RA more than 20 years ago. Both patients received biological treatment during our investigation. As expected, neither IFN-γ nor IL-4 mRNA expression levels were found elevated in the BCG group (Figure 1). Peptide stimulation induced neither FoxP3 nor IL-17 expressions in any of the study groups (data not shown).

### Testing of Different Combinations of Selected Genes As Potential Biomarkers to Differentiate Active TB from LTBI

**Figure 2:**
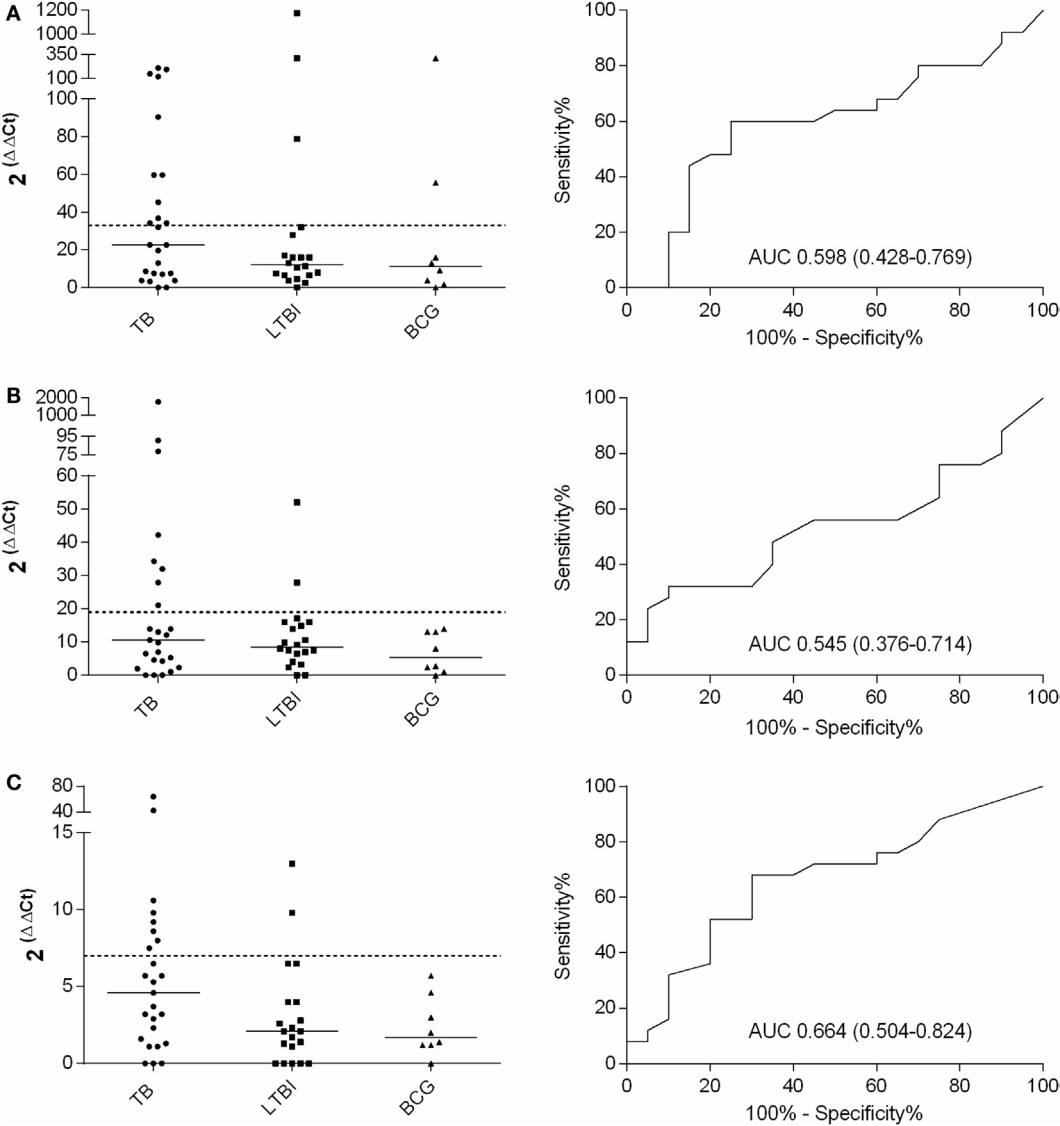
**Fold increases in IFN-γ (A), IL-17 (B), and IL-4 (C) mRNA expression after PPD stimulation and the respective ROC curves with AUCs (CI 95%)**. TB, *n* = 25; LTBI, *n* = 20; and BCG, *n* = 8. The cutoff values for each assay are shown with dashed lines and the medians of the gene expression levels for each group with horizontal bars.

For the final assessment, *IFN-*γ, *IL-17*, *IL-4*, and *FoxP3* genes and PPD stimulation were selected. Altogether 25, 20, and 8 samples from TB, LTBI, and BCG groups were analyzed, respectively. A comparison of the *FoxP3* gene expression between the TB and LTBI groups showed highly overlapping levels and no differences in the medians (data not shown). After PPD stimulation, in the TB group, the IFN-γ, IL-17, and IL-4 mRNA expression levels were higher than in the LTBI group (Figures 2A–C), yet these differences did not prove statistically significant (Table 3). The cutoff levels for the IFN-γ, IL-17, and IL-4 assays to discriminate the TB from the LTBI group were assessed with ROC curve and AUC analyses (Figures 2A–C; Table 3). The cutoffs were set at the levels with at least 85% specificity. In the BCG group, two subjects reacted over the cutoff in the IFN-γ expression assay, but none in the IL-17 or IL-4 expression. Altogether, 11/25, 8/25, and 8/25 subject from the TB group and 3/20, 2/20, and 2/20 from the LTBI groups produced positive results for the IFN-γ, IL-17, and IL-4 expression levels, respectively. Next, the discriminative ability of the cumulative expression of IFN-γ, IL-17, and IL-4 was analyzed (Table 3). No statistically significant differences between the groups were observed with any of the combinations tested. However, a sensitivity of 48% and a specificity of 85% were achieved when calculating the cumulative expression of IFN-γ, IL-17, and IL-4 or IFN-γ and IL-17 mRNAs (Table 3). Alternatively, any positive result in at least one test of IFN-γ, IL-17, or IL-4 resulted in a sensitivity of 64% and a specificity of 75%. Positive results in at least in IFN-γ or IL-4 produced a sensitivity of 60% and the specificity of 80%. The results of all the tested combinations are shown in Table 3.

**Table 3:** **Cytokine gene expression levels and their combinations measured from PPD-stimulated PBMCs to discriminate patients with active TB from LTBI**.

Cytokine combination	Positive active TB (*n* = 25)	Positive LTBI (*n* = 20)	Sens. (%)	Spec. (%)	AUC (CI 95%)	*p*
IFN-γ	11	3	44	85	0.598 (0.426–0.770)	0.263
IL-17	8	2	32	90	0.534 (0.363–0.705)	0.698
IL-4	8	2	32	90	0.662 (0.500–0.824)	0.064
IFN-γ + IL-17 + IL-4	12	3	48	85	0.586 (0.414–0.758)	0.326
IFN-γ + IL-17	12	3	48	85	0.551 (0.376–0.726)	0.560
IFN-γ + IL-4	9	3	36	80	0.623 (0.452–0.794)	0.160
IL4 + IL-17	11	3	44	85	0.594 (0.426–0.762)	0.283
IFN-γ or IL-17 or IL-4	16	5	64	75	n/a	n/a
IFN-γ or IL-17	13	4	52	80	n/a	n/a
IFN-γ or IL-4	15	4	60	80	n/a	n/a
IL-17 or IL-4	12	4	48	80	n/a	n/a

### Various Combinations of Selected Genes As Potential Biomarkers to Differentiate Active TB from LTBI in Patients with RA or with Miscellaneous Inflammatory Conditions

**Figure 3:**
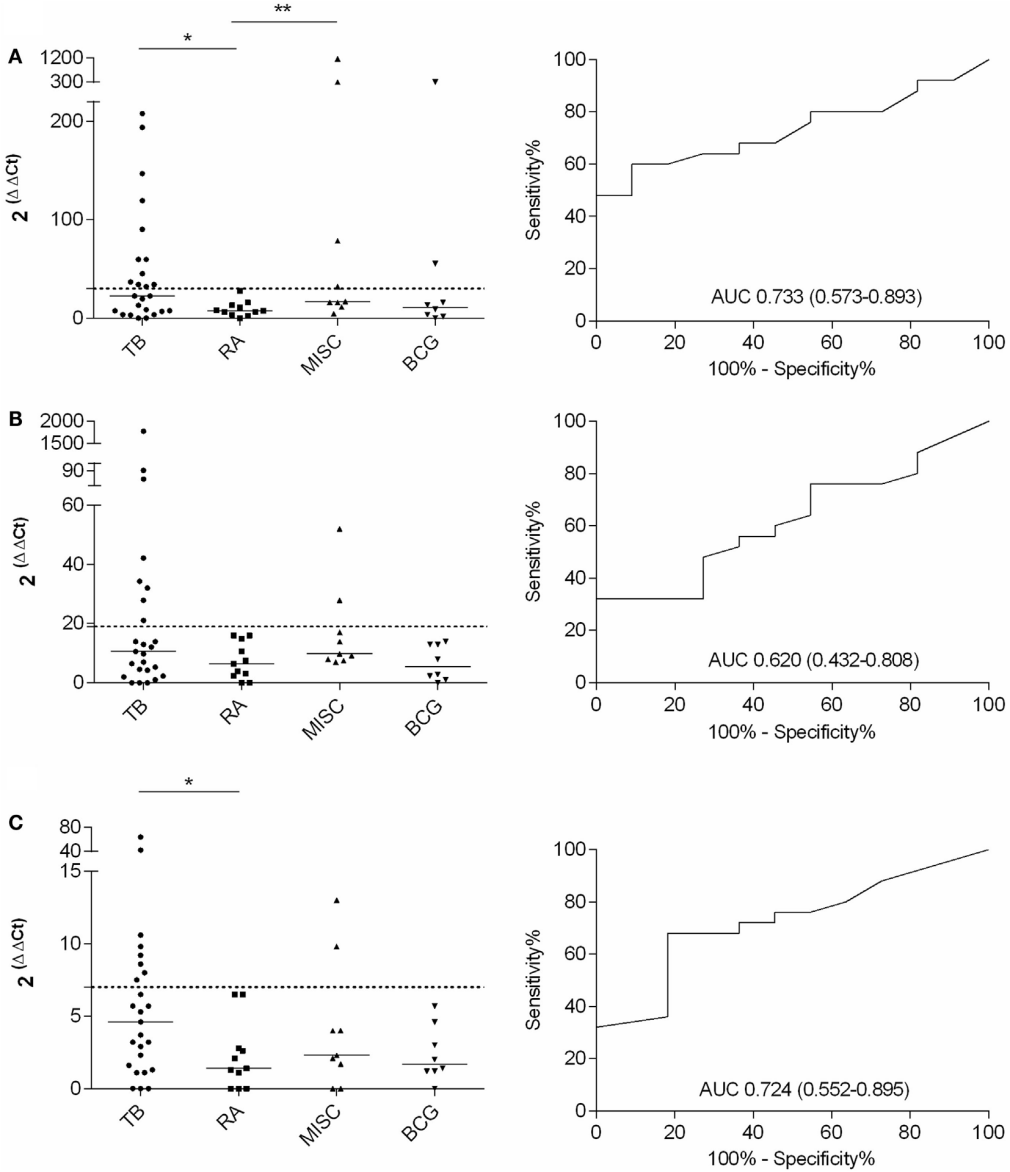
**Fold increase in IFN-γ, IL-17, and IL-4 mRNA expression after PPD stimulation and the respective ROC curves with AUCs (CI 95%) calculated with TB and RA groups**. TB, *n* = 25; RA, *n* = 11; MISC, *n* = 9; and BCG, *n* = 8. **(A)** IFN-γ, **(B)** IL-17, and **(C)** IL-4. Dashed lines represent the cutoff for each assay and the horizontal bars represent the medians of the gene expression level for each group.

Quantitative expression of IFN-γ, IL-17, and IL-4 mRNAs to differentiate active TB from LTBI in an RA or MISC groups was investigated. A significant difference in IFN-γ (*p* < 0.05) and IL-4 (*p* < 0.05) mRNA expression levels was found between the TB and the RA groups (Figures 3A–C; Table 4). In IL-17 mRNA analysis, the difference between TB and RA groups was not statistically significant (*p* = 0.264), yet there was a positive trend toward discrimination (Table 4). When the MISC and the TB group were compared, neither the differences in the cytokine levels or their combinations reached statistical significance (Table 4). In the MISC group, the highest IFN-γ mRNA expression levels were found in patients with tonsillitis and unknown skin infection. IL-17 mRNA expression levels were increased in patients with myiasis and ankylosing spondylitis, and IL-4 in patients with vasculitis and unknown skin infection.

**Table 4:** **Cytokine gene expression levels and their combinations measured from PPD-stimulated PBMCs to discriminate patients with active TB from LTBI with RA**.

Cytokine combination	Positive Active TB (*n* = 25)	Positive LTBI + RA (*n* = 11)	Sens. (%)	Spec. (%)	AUC (CI 95%)	*p*
IFN-γ	12	0	48	100	0.733 (0.573–0.893)	<0.05*
IL-17	8	0	32	100	0.620 (0.432–0.808)	0.264
IL-4	8	0	32	100	0.724 (0.552–0.895)	<0.05*
IFN-γ + IL-17 + IL-4	14	0	56	100	0.720 (0.556–0.884)	<0.05*
IFN-γ + IL-17	14	0	56	100	0.675 (0.501–0.848)	0.102
IFN-γ + IL-4	14	0	56	100	0.758 (0.603–0.914)	<0.05*
IL-4 + IL-17	11	0	44	100	0.693 (0.518–0.867)	0.069
IFN-γ or IL-17 or IL-4	16	0	64	100	n/a	n/a
IFN-γ or IL-17	13	0	52	100	n/a	n/a
IFN-γ or IL-4	16	0	64	100	n/a	n/a
IL-17 or IL-4	12	0	48	100	n/a	n/a

*p < 0.05 was considered significant.

The ROC curves with AUCs for the IFN-γ, IL-17, and IL-4 mRNA assays were constructed with the TB and RA groups, and the cutoffs were assessed to such a level where all the patients in the RA group remained below the cutoff line (Figure 3). With this approach, 48, 32, and 32% sensitivities were achieved in IFN-γ, IL-17, and IL-4 assays, respectively, for TB and RA groups. When the cumulative mRNA expressions were calculated, improved sensitivity up to 56% was found in most of the combinations (Table 4). Finally, the analysis of any positive result in at least one of the tests of IFN-γ, IL-17, or IL-4 resulted in the sensitivity of 64% with 100% specificity.

## Discussion

Distinguishing active TB from LTBI, particularly, in patients with underlying inflammatory conditions that may resemble the clinical presentation of TB, remains an important clinical challenge. It is especially demanding in patients with a history of potential exposure to TB who currently run fever of unknown cause and show elevated inflammatory markers, yet lack bacteriological confirmation of TB. Smear microscopy may not be sensitive, and culture takes too much time detecting *Mtb*. Nucleic acid amplification methods, even if sensitive, are expensive and not widely available. The IGRA techniques, although applicable to *ex vivo* detection of sensitized T lymphocytes, are not very useful in diagnosing active TB. Furthermore, at present, biomarkers or a diagnostic test does not exist to predict disease activation in immunosuppressed individuals. Here, we present the investigation of the quantitative expressions of IFN-γ, IL-17, and IL-4, the cytokines presenting the major pathways of CD4^+^ cells differentiation after antigenic stimulus. In our study, the combined quantitative analysis of genes coding for IFN-γ, IL-17, and IL-4 proteins from the PPD-stimulated PBMCs distinguished active TB from LTBI with concomitant miscellaneous inflammatory conditions only moderately. When each cytokine was analyzed independently, the differences between the groups were not found statistically significant. The cumulative expression of IFN-γ, IL-17, and IL-4, or IFN-γ and IL-17, resulted in 48% sensitivity with 85% specificity remaining below the desired specifications for a clinical test. Alternatively, when any of the tests for IFN-γ, IL-17, or IL-4 was over the cutoff, the assay showed a sensitivity of 64% and specificity of 75%. When disease stage discriminating ability of IFN-γ, IL-17, and IL-4 were tested with a more homogeneous LTBI group, namely the RA group, the combined analysis of IFN-γ, IL-17, and IL-4 or IFN-γ and IL-4 resulted in 64% sensitivity (*p* < 0.05) with 100% specificity. Thus, scrutinizing the patients groups into more homogenous ones, we were able to improve the discrimination power of the cytokine measurement. Nonetheless, we want to underline the inevitable fact that in the real clinical situation, when the diagnosis of a patient has remained unclear, the clinician is expecting to get from a laboratory a definitive interpretation regarding each individual sample, but not statistics. In our opinion, proper diagnostic method should be robust enough to categorize patients into groups. Statistical significance between the groups is in fact not sufficient to qualify any method as a diagnostic tool.

A feature that adds weight to our study is the enrollment of LTBI patients with other underlying diseases, such as rheumatoid diseases. In many previous studies (14, 16, 24), LTBI group has been represented by household contacts (HHC). Selecting, instead, LTBI group members among subjects with underlying inflammatory conditions is expected to be more challenging from the clinical standpoint. These patients, similar to those with active TB, have a systemic inflammatory condition, which indicates *a priori* a misbalanced cytokine production (25, 26).

The usefulness of a variety of immunological biomarkers in TB infection has been extensively investigated during last decades. The controversy regarding the applicability of any marker may partly be due to a great variety of methodologies. In some studies, cell subpopulation frequencies were measured (27), while in others cell phenotypical or functional profiles were studied (28), and yet in some others, cytokine production levels were measured with ELISA (24) or (18), like in our study, gene expression levels coding certain proteins were quantified.

One of the limitations of the study includes the relatively small cohorts of tested individuals and the great variability in the spectrum of underlying diseases in the MISC group. The degree of immunosuppression varied between the patients. However, none of the patients presented such a degree of immunosuppression that it would have prevented immunoreactivity in IGRA tests.

In many studies (including ours), the duration of anti-TB therapy in TB patients is variable, which may be a confounding factor. We attempted to standardize the duration of the anti-TB therapy by only enrolling patients having received treatment for no longer than 2 weeks (median: 7 days, range: 0–14 days).

Limited repertoire of the tested genes is another limitation of our study, but we aimed to test only the mainstream markers of T cell differentiation.

In real practice, LTBI patients requiring differential diagnostics from active TB are a highly heterogenic group. Each condition within this group may present with a different degree of cytokine misbalance. LTBI group can comprise either HHC (practically healthy) or patients with RA with highly dysregulated cytokine and chemokine production. An additional challenge in trying to find sustainable immunological signatures lies in the nature of the TB infection, which is by no means a homogenous condition. Studies of cynomolgus macaques have shown that there is a wide spectrum of overlapping stages of infection presenting with different degrees of tissue damage and immune responses (29). According to this novel understanding, active TB is a continuum of LTBI or subclinical TB. Therefore, it appears imperative that future search for new immunological signatures be linked with the results of modern imaging tests (e.g., positron emission tomography) or other imaging techniques estimating the severity or current stage of lung pathology (30). Infection stage differentiation will most probably require simultaneous analysis of multiple markers underscoring the regulation of various genes (genomics) or protein expression profiles (proteomics) (18, 24, 31). These findings should be linked, e.g., to the types of granulomas and the location and viability of the bacillus in different outcomes of TB.

Contrary to our findings, IFN-γ mRNA expression levels have been reported to be significantly higher in LTBI than TB patients, when the cells are stimulated with the recombinant ESAT-6 (18). The IFN-γ mRNA expression levels have been correlated with the production of the corresponding protein by the cells stimulated in the QuantiFeron^®^ tubes (32). In accordance with our findings, ESAT-6 and CFP-10 protein stimulation has not proved as efficient as the PPD stimulation (11, 24, 33). Our study showed that expression levels of the major genes associated with T-cell differentiation only moderately discriminated active TB from LTBI. As a stimulus, we used PPD for the reason that, in active TB, bacterial load is higher and, in replicating bacteria, antigen expression is more variable than, e.g., by dormant bacteria of LTBI (34). Higher variability of antigens better diversifies immune response resulting in proliferation of T-cell clones with a wider receptor repertoire. Stimulation with PPD therefore better targets all reactive T-cell clones than, e.g., stimulation with only ESAT-6 or CFP-10.

An increased IL-17 mRNA expression has been described in mononuclear cells from pleural fluid of patients with tuberculous pleurisy (21). Compared to the healthy controls, increased IL-17 mRNA expression levels have also been found from TB patients’ unstimulated PBMCs (33). In our study, antigen-stimulated IL-17 mRNA expression was associated with active TB, rather than with LTBI.

Variable expressions of IL-4 and IL4δ2 mRNAs have been reported in the groups of active TB, LTBI, or HHCs from unstimulated cells (13, 14). Furthermore, the ratios of IL-4 to IL-4δ2 or IFN-γ of the mRNA expression levels have been suggested as a cure indicator (13, 16). We also detected elevated IL-4 mRNA expression levels in patients with active TB.

The IL-4δ2 gene was expressed at very low levels even after stimulation. Therefore, it was omitted from further analysis.

Rises in FoxP3 mRNA expression and expansion of Treg cells have also reported to be more common in patients with active TB than in those with the LTBI (11, 18). Our data did not reveal differences between the FoxP3 mRNA expression levels.

As a conclusion, we chose to explore the potential of combined quantitative expression profiling of some main markers of T cell activation and differentiation, IFN-γ, IL-17, and IL-4 mRNA, to discriminate between active TB and LTBI in clinical patient groups. A clinical setting is demanding as such, yet these are the patient groups representing the greatest challenge in clinical practice.

Presented results suggest that the undertaken approach seems to have only a moderate diagnostic value to qualify for routine use. Alternative approaches, probably based on pathogen-related biomarkers, should be exploited.

## Author Contributions

LS: designed the study, collected clinical samples, performed the analyses, interpreted the data, and wrote the first draft. AKa: collected clinical samples and applied for the ethical clearances. AKn, LP, RK, and HV: collected clinical samples. TT: designed the study, applied for the ethical clearances, and wrote the first draft. All authors contributed to the manuscript preparation, read, approved, and accepted the final version.

## Conflict of Interest Statement

The authors declare that they have no conflict of interests. Preliminary results of this study were reported as a poster at annual meeting of Nordic Society of Clinical microbiology and Infectious Diseases, 2013.
